# Role of Thioredoxin 1 in Impaired Renal Sodium Excretion of *hD*_*5*_ *R* ^*F173L*^ Transgenic Mice

**DOI:** 10.1161/JAHA.119.012192

**Published:** 2019-04-06

**Authors:** Shaoxiong Wang, Xiaorong Tan, Peng Chen, Shuo Zheng, Hongmei Ren, Jin Cai, Lin Zhou, Pedro A. Jose, Jian Yang, Chunyu Zeng

**Affiliations:** ^1^ Department of Cardiology Daping Hospital Army Medical University of PLA Chongqing P.R. China; ^2^ Division of Renal Disease & Hypertension Departments of Medicine and Pharmacology/Physiology The George Washington University School of Medicine and Health Sciences Washington DC; ^3^ Department of Clinical Nutrition The Third Affiliated Hospital of Chongqing Medical University Chongqing P.R. China

**Keywords:** dopamine D_5_ receptor, *hD_5_R^F173L^*‐TG, hypertension, kidney, reactive oxygen species, thioredoxin 1, High Blood Pressure, Hypertension

## Abstract

**Background:**

Dopamine D_5_ receptor (D_5_R) plays an important role in the maintenance of blood pressure by regulating renal sodium transport. Our previous study found that human D_5_R mutant F173L transgenic (*hD*_*5*_ *R* ^*F173L*^‐TG) mice are hypertensive. In the present study, we aimed to investigate the mechanisms causing this renal D_5_R dysfunction in *hD*_*5*_ *R* ^*F173L*^‐TG mice.

**Methods and Results:**

Compared with wild‐type D_5_R‐TG (*hD*_*5*_ *R* ^*WT*^‐TG) mice, *hD*_*5*_ *R* ^*F173L*^‐TG mice have higher blood pressure, lower basal urine flow and sodium excretion, and impaired agonist‐mediated natriuresis and diuresis. Enhanced reactive oxygen species production in *hD*_*5*_ *R* ^*F173L*^‐TG mice is caused, in part, by decreased expression of antioxidant enzymes, including thioredoxin 1 (Trx1). Na^+^‐K^+^‐ATPase activity is increased in mouse renal proximal tubule cells transfected with *hD*_*5*_ *R* ^*F173L*^, but is normalized by treatment with exogenous recombinant human Trx1 protein. Regulation of Trx1 by D_5_R occurs by the phospholipase C/ protein kinase C (PKC) pathway because upregulation of Trx1 expression by D_5_R does not occur in renal proximal tubule cells from D_1_R knockout mice in the presence of a phospholipase C or PKC inhibitor. Fenoldopam, a D_1_R and D_5_R agonist, stimulates PKC activity in primary renal proximal tubule cells of *hD5R* ^*WT*^ *‐*TG mice, but not in those of *hD*_*5*_ *R* ^*F173L*^‐TG mice. Hyperphosphorylation of hD_5_R^F173L^ and its dissociation from Gαs and Gαq are associated with impairment of D_5_R‐mediated inhibition of Na^+^‐K^+^‐ATPase activity in *hD*_*5*_ *R* ^*F173L*^‐TG mice.

**Conclusions:**

These suggest that *hD*_*5*_ *R* ^*F173L*^ increases blood pressure, in part, by decreasing renal Trx1 expression and increasing reactive oxygen species production. Hyperphosphorylation of hD_5_R^F173L^, with its dissociation from Gαs and Gαq, is the key factor in impaired D_5_R function of *hD*_*5*_ *R* ^*F173L*^‐TG mice.

**Clinical Perspective**

**What Is New?**

- *hD*_*5*_ *R* ^*F173L*^‐TG mice, compared with wild‐type mice, are hypertensive with lower basal urine flow and sodium excretion, and impaired diuretic and natriuretic responses to D_1_‐like receptor agonists.
- Decreased thioredoxin 1 expression and function mediates the impaired renal urine flow and sodium excretion in *hD5R* ^F173L^‐TG mice.
- The hyperphosphorylation of hD_5_R^F173L^, with its dissociation from Gαs and Gαq, is the key factor in the impaired D_5_R function in *hD*_*5*_ *R* ^*F173L*^‐TG mice.

**What Are the Clinical Implications?**

- The present study reinforces the role of dopamine D_5_ receptor in hypertension and shows the role of thioredoxin 1 in the impaired sodium excretion and increased blood pressure in *hD*_*5*_ *R* ^*F173L*^‐TG mice.
- The aberrant D_5_R/phospholipase C/protein kinase C (PKC)/thioredoxin 1 signaling may be involved in the pathogenesis of genetic hypertension.

## Introduction

Essential hypertension is one of the most common health risk factors in both developed and developing countries. In 2015, global age‐standardized prevalence of elevated blood pressure was 24.1% in men and 20.1% in women.^1^ High systolic blood pressure was the third‐leading health risk and cause of associated deaths in the United States in 2016. Reductions in death rates arising from cardiovascular disease have been related, in part, to reductions in systolic blood pressure.^2^

The kidney is the major organ involved in the long‐term control of blood pressure, in part by maintaining sodium homeostasis.^3^ Humans with polygenic essential hypertension exhibit enhanced sodium transport in the proximal tubule of the kidney, which is regulated by numerous hormones and humoral factors, such as dopamine and its receptors.^4^, ^5^ Dopamine receptors have been classified into 2 subtypes: D_1_‐like receptors, which include D_1_R and D_5_R, and D_2_‐like receptors, which include D_2_R, D_3_R, and D_4_R.^4^, ^5^, ^6^ D_5_R is expressed in much of the mammalian kidney, specifically in the proximal tubule, in the thick ascending limb of Henle, in the distal convoluted tubule, and in the cortical collecting duct.^7^, ^8^, ^9^ Compared with other dopamine receptors, D_5_R has the highest affinity for dopamine and exhibits constitutive activity, which can be further activated in the absence or presence of low concentrations of endogenous agonists.^9^, ^10^, ^11^

D_5_R, as do other dopamine receptor subtypes, plays a vital role in the maintenance of normal body sodium and blood pressure by its own action and also through interactions with other dopamine receptors and G‐protein‐coupled receptors.^4^, ^5^, ^6^, ^7^, ^8^, ^9^ The human D_5_R gene *DRD5* locus, 4p15.1 to 16.1, and its pseudogenes, 1q21.1 and 2p11.1‐p11.2, have been associated with human essential hypertension.^12^, ^13^ Disruption of *Drd5* in mice results in hypertension that is aggravated by a high‐salt diet.^14^, ^15^, ^16^ Renal expression of the angiotensin II type 1 receptor and renal sodium transporters are also increased in *Drd5* ^−/−^ mice.^14^, ^15^, ^16^ In addition, *Drd5* ^−/−^ mice exhibit increased oxidative stress.^17^, ^18^ D_5_R decreases reactive oxygen species (ROS) production by inhibiting the expression and activity of phospholipase D and nicotinamide adenine dinucleotide phosphate oxidase and by upregulating heme oxygenase‐1.^17^, ^18^, ^19^ α/β hydroxylase 1 could also be involved in D_5_R‐mediated regulation of ROS production.^20^

Humans carry single‐nucleotide polymorphisms in the *DRD5* gene, some of which confer diminished D_5_R function and abnormal coupling with adenylyl cyclase.^21^, ^22^, ^23^ The human D_5_R F173L (*hD*_*5*_ *R* ^*F173L*^) mutation markedly impairs stimulation of cAMP production.^17^, ^24^ To investigate the role of *hD*_*5*_ *R* ^*F173L*^ in the elaboration of hypertension, we generated *hD*_*5*_ *R* ^*F173L*^ transgenic (*hD*_*5*_ *R* ^*F173L*^‐TG) and *hD*_*5*_ *R* wild‐type transgenic (*hD*_*5*_ *R* ^*WT*^‐TG) mice. Our previous study showed that only *hD*_*5*_ *R* ^*F173L*^‐TG mice exhibited hypertension.^24^ However, the mechanisms causing renal D_5_R dysfunction in these *hD*_*5*_ *R* ^*F173L*^‐TG mice have not been clear. The present study investigated the role of thioredoxin 1 (Trx1), an antioxidant that interacts with other antioxidant proteins, in the establishment of oxidative stress in mouse renal proximal tubule (RPT) cells and transgenic mice carrying *hD*_*5*_ *R* ^*F173L*^, with *hD*_*5*_ *R* ^*WT*^ in RPT cells and transgenic mice serving as controls.

## Methods

The data, analytic methods, and study materials will be made available to other researchers for purposes of reproducing the results or replicating the procedure.

### Generation of *D*_*5*_ *R* ^*F173L*^ Transgenic Mice

*hD*_*5*_ *R* ^*F173L*^‐TG and *hD*_*5*_ *R* ^*WT*^‐TG mice were generated by microinjection of pcDNA2*hD*_*5*_ *R* ^*F173L*^ and pcDNA2*hD*_*5*_ *R* ^*WT*^ into oocytes.^24^ The difference of the wild‐type allele and mutant allele in the transgenic mice is shown in Figure S1A. Both of these transgenic lines were maintained in our animal facility by backcrossing them with C57BL/6 mice (Charles River, St‐Constant, Quebec, Canada). Genotypes of transgenic founders and their offspring were identified using polymerase chain reaction (PCR) with transgene‐specific primers. Relative expression of D_5_R protein was determined by immunoblotting.

### Mouse Experiments

All procedures used in this study were approved by the Third Military Medical University Animal Use and Care Committee. All experiments conformed to the guidelines for the ethical use of animals, and all efforts were made to minimize animal suffering and reduce the number of animals used.

To study the effects of D_5_R on renal function in *hD*_*5*_ *R* ^*F173L*^‐TG mice, we compared the effect of fenoldopam, a D_1_‐like receptor agonist,^14^, ^17^ on urinary sodium and water excretion of 4‐month‐old male *hD*_*5*_^*WT*^‐TG and *hD*_*5*_ *R* ^*F173L*^‐TG mice. Mice were initially anesthetized by intraperitoneal injection of pentobarbital (50 mg/kg) and maintained under anesthesia by intravenous infusion of pentobarbital (0.8 mg/100 g body weight/h), as reported in our previous studies.^25^, ^26^ Anesthetized mice were placed on a heated blanket to maintain body temperature at ≈37°C measured rectally and were tracheotomized (PE50). The left external jugular vein was catheterized (PE10) for fluid administration whereas the left carotid artery was catheterized (PE10) for monitoring of blood pressure. Urine was collected by a suprapubic cystostomy. Fluid losses during surgery (≈60 minutes) were replenished with 5% albumin in normal saline at 1% body weight over 30 minutes. After a 120‐minute equilibration period, urine was collected for 40 minutes per period for a total of 5 collection periods. Urinary sodium concentration was measured using an electrolyte analyzer (HC988; Histrong Medical, Shenzhen, China) by the ion‐selective electrode method. A noninvasive method (MODEL MK‐2000; Muromachi Kikai Co. Ltd, Tokyo, Japan) was also used to measure blood pressure on the tails of conscious unanesthetized *hD*_*5*_ *R* ^*F173L*^‐TG and *hD*_*5*_ *R* ^*WT*^ *‐TG* mice.

### Mouse RPT Cell Experiments

The mouse RPT cells used in these experiments were originally provided by Dr Ulrich Hopfer of the Case Western Reserve University School of Medicine.^27^ The *hD*_*5*_ *R* ^*F173L*^ and *hD*_*5*_ *R* ^*WT*^ plasmids were transfected into mouse RPT cells using Lipofectamine 2000 (Invitrogen, Carlsbad, CA). RPT cells (90% confluence) were collected and homogenized in ice‐cold lysis buffer (20 mmol/L of Tris‐HCl, pH 7.4; 2 mmol/L of EDTA, pH 8.0; 2 mmol/L of EGTA; 100 mmol/L of NaCl; 10 μg/mL of leupeptin; 10 μg/mL of aprotinin; 2 mmol/L of phenylmethylsulfonyl fluoride; and 1% Nonidet P‐40). Homogenates were then sonicated for 20 seconds, kept on ice for 1 hour, and centrifuged at 16 000*g* for 30 minutes. All samples were stored at −70°C until use.

### Primary Culture of Mouse RPT Cells

RPT cells were isolated from kidneys of *hD*_*5*_ *R* ^*F173L*^‐TG mice, *hD*_*5*_ *R* ^*WT*^‐TG mice, and D_1_R knockout mice according to the methods of a previously published study.^28^ RPT cells from D_1_R knockout mice were also used for comparisons to eliminate the confounding effect of the other D_1_‐like receptor, D_1_R,^4^, ^5^, ^6^, ^7^, ^8^, ^9^ because there are no commercially available agonists or antagonists that can distinguish the activities of D_1_R from those of D_5_R. Briefly, immediately after harvesting the kidneys, renal cortices were collected and minced on ice‐cold plates. Minced tissues were digested for 15 minutes with 0.75 mg/mL of collagenase type II at 37°C in HBSS. Digestion was stopped by mixing the digests with ice‐cold 10% FBS (Gibco, Life Technologies, Carlsbad, CA). The suspension was then sequentially filtered through 2 sieves (250 and 70 μm). Subsequently, cells were washed with ice‐cold HBSS and serum‐free DMEM/F‐12 medium (Gibco, Life Technologies). Mouse RPT cells were purified by centrifugation for 10 minutes at 2000*g* in 32% Percoll at 4°C. The pellet was collected and washed twice with ice‐cold serum‐free DMEM/F‐12 medium. Finally, mouse RPT cells were plated onto collagen‐coated dishes and cultured in DMEM/F‐12 medium supplemented with 5 mg/mL of transferrin, 5 mg/mL of insulin, 0.05 mmol/L of hydrocortisone, and 50 mmol/L of ascorbic acid.

### Dihydroethidium Staining

Superoxide production in kidney was quantified using the fluorescent dye, dihydroethidium. Frozen sections of mouse kidneys were stained with dihydroethidium (10^−5^ mol/L) for 20 minutes. After washing, images were taken using a fluorescence microscope (ECLIPSE Ti; Nikon, Tokyo, Japan) with excitation wavelength at 490 nm and emission wavelength at 590 nm. All sections were processed under the same conditions. Settings for image acquisition were identical for all sections. Dihydroethidium fluorescence intensity was quantified using ImageJ software (National Institutes of Health, Bethesda, MD).

### Measurement of Malondialdehyde Levels

Lipid peroxidation was determined by measuring malondialdehyde (MDA) levels, using the Lipid Peroxidation MDA Assay Kit (Beyotime Biotech, Nanjing, China). Quantification is based on the formation of thiobarbituric acid reactive substances. After obtaining samples of blood, they were centrifugated at 367*g* for 15 minutes. The serum was used to measure MDA levels. Supernatant (100 μL) was transferred into a tube containing 1.4 mL of 0.37% thiobarbituric acid. The reaction mixture was vortexed and incubated at 95°C for 15 minutes. After cooling, values were read spectrophotometrically at 532 nm. Levels of MDA are expressed as μmol/L.

### Real‐Time Quantitative PCR

Renal tissues were obtained from 3‐month‐old *hD*_*5*_ *R* ^*F173L*^‐TG and *hD*_*5*_ *R* ^*WT*^‐TG mice. Total RNA was isolated using TRIzol reagent following the procedures. RNA content was measured spectrophotometrically (DU800; Beckman Coulter, Brea, CA). Reverse‐transcription reactions were performed with 1 μg of total RNA as a template. Real‐time quantitative PCR was performed after mixing cDNA with SYBR GreenER qPCR SuperMix Universal (Invitrogen). For amplification, 2 μL of cDNA was used per 25‐μL final reaction volume. Mouse *Trx1*, superoxide dismutase‐1 (*Sod‐1*), glutathione peroxidase‐1 (*Gpx‐1*), catalase (*Cat*), and peroxisome proliferator‐activated receptor γ (*Pparγ*) gene‐specific primers used for PCR are shown in Table S1.

### Immunoblotting

Total protein (50 μg) was separated by electrophoresis on 10% or 15% SDS‐PAGs and transferred onto nitrocellulose membranes. Anti‐Trx1 antibody (1:500 dilution; Cell Signaling Technology, Danvers, MA) or D_5_R antibody (1:500 dilution; Santa Cruz Biotechnology, Santa Cruz, CA) were used as primary antibody. Proteins were visualized using an Odyssey scanner (Li‐COR, Lincoln, NE). Densities of bands were normalized against that of glyceraldehyde‐3‐phosphate dehydrogenase.

### Na^+^‐K^+^‐ATPase Activity Assay

We measured Na^+^‐K^+^‐ATPase activity of RPT cells treated with recombinant human Trx1 protein (T8690; Sigma‐Aldrich, St. Louis, MO) and transfected with either *hD*_*5*_ *R* ^*F173L*^ or *hD*_*5*_ *R* ^*WT*^ plasmid. Na^+^‐K^+^‐ATPase activity in the crude membrane fraction was measured using ouabain to inhibit Na^+^‐K^+^‐ATPase activity, as previously described.^29^, ^30^ Na^+^‐K^+^‐ATPase activity was calculated as the difference between total ATPase activity and ouabain‐insensitive ATPase activity and was then corrected according to cell protein content. All experiments were performed simultaneously with controls.

### Protein Kinase C Activity Assay

Protein kinase C (PKC) activity was determined using a nonradioactive method.^31^ Briefly, RPT cells were lysed in a modified PKC extraction buffer (25 mmol/L of Tris, 0.05% Triton X‐100, 10 mmol/L of β‐mercaptoethanol, and protease and phosphatase inhibitors). After protein extraction and quantification, equal volumes of protein extract were used in each PKC reaction following the protocol in the PepTag assay for nonradioactive detection of PKC (#V5330; Promega, Madison, WI). Protein samples were then incubated with a positively charged fluorescent PKC‐specific peptide for 30 minutes and separated on agarose gels. The phosphorylated negatively charged peptide was separated from the nonphosphorylated positively charged peptides and visualized under ultraviolet light. The resulting bands were quantified by densitometry and normalized to controls. PKC activities in the samples were normalized according to their protein concentrations.

### Immunoprecipitation

Renal cortices were homogenized in ice‐cold lysis buffer for 1 hour and centrifuged at 12 000*g* for 15 minutes. After measuring protein concentrations, equal amounts of renal homogenates (500 μg of protein/mL of supernatant) were incubated with affinity‐purified anti‐D_5_R antibody (3 μg/mL; D_5_R/Gαq and D_5_R/Gαs coimmunoprecipitation) for 1 hour and with protein‐G agarose at 4°C for 12 hours. Immunoprecipitates were subjected to immunoblotting with anti‐Gαq or anti‐Gαs antibody (Santa Cruz Biotechnology). To determine the specificity of the bands found on the immunoblots, immunoglobulin G (negative control) and anti‐Gαq or ‐Gαs antibodies (positive controls) were used for immunoprecipitation instead of the D_5_R antibody.

To determine level of D_5_R phosphorylation, supernatants were immunoprecipitated with anti‐D_5_R antibody. Immunoprecipitates were then subjected to immunoblotting with antiphosphoserine or antiphosphothreonine antibodies (Santa Cruz Biotechnology).

### Statistical Analysis

All values are expressed as the mean±SEM. Data were analyzed by performing 1‐way ANOVA for comparisons within groups (or 2‐tailed unpaired *t* tests when only 2 groups were compared), and 2‐way ANOVA for comparison of 2 variables between groups with Bonferroni's correction using GraphPad Prism software (GraphPad Software, La Jolla, CA). Values of *P*<0.05 were considered significant.

## Results

### Increased Blood Pressure and Decreased Urine Sodium Excretion in *hD*_*5*_ *R* ^*F173L*^‐TG Mice

**Figure 1:**
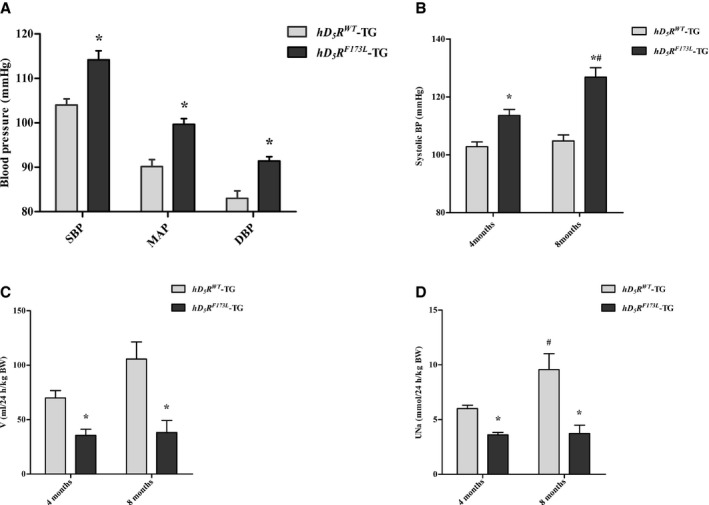
Blood pressure and renal function in *hD*_*5*_ *R* ^*F173L*^ ‐TG mice. **A**, SBP, DBP, and MAP measured from the carotid artery in anesthetized 4‐month‐old *hD*_*5*_ *R* ^*WT*^‐TG and *hD*_*5*_ *R* ^*F173L*^‐TG mice (*P<0.05 compared with *hD*_*5*_ *R* ^*WT*^‐TG mice; n=5). **B**, SBP measured by the tail‐cuff method in conscious, nonanesthetized 4‐ and 8‐month‐old *hD*_*5*_ *R* ^*WT*^‐TG and *hD*_*5*_ *R* ^*F173L*^‐TG mice (*P<0.05 compared with *hD*_*5*_ *R* ^*WT*^‐TG mice; ^#^ *P*<0.05 compared with others; n=5). **C** and **D**, Urine volume (**C**) and UNa (**D**) in 4‐ and 8‐month‐old *hD*_*5*_ *R* ^*WT*^‐TG and *hD*_*5*_ *R* ^*F173L*^‐TG mice (*P<0.05 compared with *hD*_*5*_ *R* ^*WT*^‐TG mice; ^#^ *P*<0.05 compared with others; n=5). BW indicates body weight; DBP, diastolic blood pressure; *hD*_*5*_ *R* ^*F173L*^ ‐TG, human dopamine D_5_ receptor mutant F173L transgenic; *hD*_*5*_ *R* ^*WT*^‐TG, human dopamine D_5_ receptor wild‐type transgenic; MAP, mean arterial blood pressure; SBP, systolic blood pressure; V, urine volume; UNa, urine sodium excretion.

To investigate the role of D_5_R in the pathogenesis of hypertension, we generated *hD*_*5*_ *R* ^*F173L*^‐TG and *hD*_*5*_ *R* ^*WT*^‐TG mice. Genotypes of transgenic founders and their offspring were confirmed by PCR (Figure S1B). There were no differences in body weight between *hD*_*5*_ *R* ^*F173L*^‐TG and *hD*_*5*_ *R* ^*WT*^‐TG mice (*hD*_*5*_ *R* ^*WT*^‐TG: 23.4±1.1 g; *hD*_*5*_ *R* ^*F173L*^‐TG: 22.7±1.2 g; n=6; 4‐month‐old). There were also no differences in sodium, water, and food intake in these 2 groups of mice (Table S2). We have previously reported that the blood pressure of *hD*_*5*_ *R* ^*F173L*^‐TG mice was higher than that of *hD*_*5*_ *R* ^*WT*^‐TG mice at 3 months of age,^24^ which was replicated in the present study in 4‐ and 8‐month‐old mice (Figure 1A and 1B). Blood pressure was measured from the carotid artery in anesthetized mice (Figure 1A) and by the tail‐cuff method in conscious mice (Figure 1B). Moreover, we further found that *hD*_*5*_ *R* ^*F173L*^‐TG mice had decreased 24‐hour urine output and sodium excretion, corrected for body weight, relative to *hD*_*5*_ *R* ^*WT*^‐TG mice (Figure 1C and 1D).

**Figure 2:**
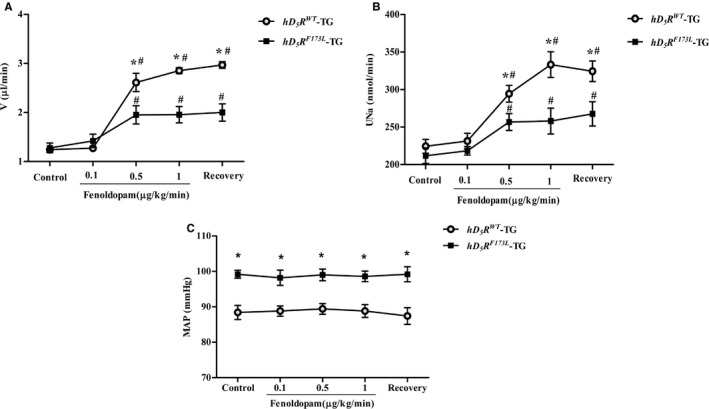
Renal function and blood pressure in fenoldopam‐treated *hD*_*5*_ *R* ^*WT*^ *‐*TG and *hD*_*5*_ *R* ^*F173L*^‐TG mice. **A** and **B**, Urine volume (**A**) and UNa (**B**) in *hD*_*5*_ *R* ^*WT*^‐TG and *hD*_*5*_ *R* ^*F173L*^‐TG mice treated with fenoldopam (*P<0.05 compared with *hD*_*5*_ *R* ^*F173L*^‐TG mice; ^#^ *P*<0.05 compared with control; n=5). **C**, MAP measured from the carotid artery in anesthetized fenoldopam‐infused *hD*_*5*_ *R* ^*WT*^‐TG and *hD*_*5*_ *R* ^*F173L*^‐TG mice (*P<0.05 compared with *hD*_*5*_ *R* ^*WT*^‐TG mice; n=5). *hD*_*5*_ *R* ^*F173L*^ ‐TG indicates human dopamine D_5_ receptor mutant F173L transgenic; *hD*_*5*_ *R* ^*WT*^‐TG, human dopamine D_5_ receptor wild‐type transgenic; MAP, mean arterial blood pressure; V, urine volume; UNa, urine sodium excretion.

To exclude the role of renal D_5_R expression bias on blood pressure, urine output, and sodium excretion, we quantified renal D_5_R protein by semiquantitative immunoblotting and found no difference in renal D_5_R protein expression between *hD*_*5*_ *R* ^*F173L*^‐TG and *hD*_*5*_ *R* ^*WT*^‐TG mice (Figure S2). Effects of fenoldopam, a D_1_R/D_5_R agonist, on urine flow and sodium excretion were then investigated. As mentioned above, there is no agonist that is selective for D_1_R, relative to D_5_R or vice versa; D_5_R has a higher affinity for dopamine than D_1_R,^9^, ^10^, ^11^ but both receptors have a similar affinity for fenoldopam, also known as SKF‐82526. Fenoldopam (0.1, 0.5, and 1.0 μg/kg body weight/min per dose administered for 40 minutes; n=5) infused into the external jugular vein increased urine flow and sodium excretion in both mouse groups, but to a lesser extent in *hD*_*5*_ *R* ^*F173L*^‐TG mice than in *hD*_*5*_ *R* ^*WT*^‐TG mice (Figure 2A and 2B), indicating impaired D_5_R function in *hD*_*5*_ *R* ^*F173L*^‐TG mice. The differential effects of fenoldopam on urine flow and sodium excretion in these 2 groups of mice were not caused by differential effects on blood pressure, because the infusion of the varying doses of fenoldopam did not affect the blood pressures in either *hD*_*5*_ *R* ^*F173L*^‐TG or *hD*_*5*_ *R* ^*WT*^‐TG mice (Figure 2C).

### Increased Oxidative Stress in *hD*_*5*_ *R* ^*F173L*^‐TG Mice

**Figure 3:**
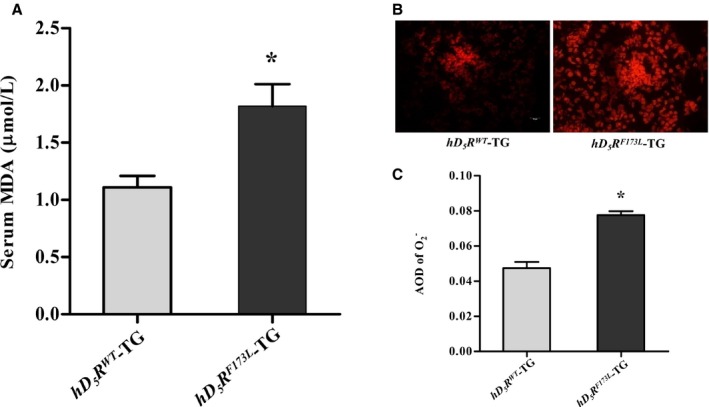
Increased oxidative stress in *hD*_*5*_ *R* ^*F173L*^ ‐TG mice. **A**, Serum MDA levels in *hD*_*5*_ *R* ^*WT*^‐TG and *hD*_*5*_ *R* ^*F173L*^‐TG mice (*P<0.05 compared with *hD*_*5*_ *R* ^*WT*^‐TG mice; n=5). **B** and **C**, Fluorescence microscopy images (**B**) and quantification (**C**) of renal ROS production in *hD*_*5*_ *R* ^*WT*^‐TG and *hD*_*5*_ *R* ^*F173L*^‐TG mice (*P<0.05 compared with *hD*_*5*_ *R* ^*WT*^‐TG mice; n=10). AOD indicates average optical density; *hD*_*5*_ *R* ^*F173L*^ *‐*TG indicates human dopamine D_5_ receptor mutant F173L transgenic; *hD*_*5*_ *R* ^*WT*^‐TG, human dopamine D_5_ receptor wild‐type transgenic; MDA, malondialdehyde; ROS, reactive oxygen species.

Previous studies have shown that D_5_R negatively regulates ROS production.^17^, ^18^, ^19^ In the present study, we compared production of renal ROS in *hD*_*5*_ *R* ^*F173L*^‐TG and *hD*_*5*_ *R* ^*WT*^‐TG mice. Results showed that serum MDA level was higher in *hD*_*5*_ *R* ^*F173L*^‐TG than in *hD*_*5*_ *R* ^*WT*^‐TG mice (Figure 3A). Moreover, renal production of ROS, measured by dihydroethidium, was enhanced in *hD*_*5*_ *R* ^*F173L*^‐TG, relative to *hD*_*5*_ *R* ^*WT*^‐TG, mice (Figure 3B and 3C), indicating increased oxidative stress in whole body and kidney of *hD*_*5*_ *R* ^*F173LT*^‐TG mice.

### Decreased Trx1 Expression and Function in *hD*_*5*_ *R* ^*F173L*^‐TG Mice

**Figure 4:**
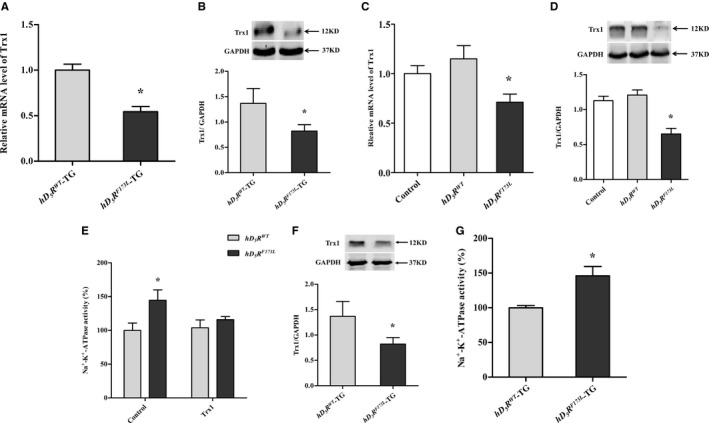
Trx1 expression and function in *hD*_*5*_ *R* ^*WT*^‐TG and *hD*_*5*_ *R* ^*F173L*^‐TG mice and *hD*_*5*_ *R* ^*WT*^‐ and *hD*_*5*_ *R* ^*F173L*^‐transfected cells. **A** and **B**, Trx1 mRNA (**A**) and protein expression (**B**) in *hD*_*5*_ *R* ^*WT*^‐TG and *hD*_*5*_ *R* ^*F173L*^‐TG mice (*P<0.05 compared with *hD*_*5*_ *R* ^*WT*^‐TG mice; n=8). **C** and **D**, Trx1 mRNA (**C**) and protein expression (**D**) in *hD*_*5*_ *R* ^*WT*^‐ and *hD*_*5*_ *R* ^*F173L*^‐transfected cells (*P<0.05 compared with control; n=6). **E**, Effect of exogenous recombinant human Trx1 protein on Na^+^‐K^+^‐ATPase activity in *hD*_*5*_ *R* ^*WT*^‐transfected and *hD*_*5*_ *R* ^*F173L*^‐transfected RPT cells. RPT cells were incubated with the recombinant human Trx1 protein (20 μg/mL) for 24 hours (*P<0.05 compared with other treatments; n=11). **F**, Trx1 protein expression in primary cultures of RPT cells from *hD*_*5*_ *R* ^*WT*^‐TG and *hD*_*5*_ *R* ^*F173L*^‐TG mice (*P<0.05 compared with *hD*_*5*_ *R* ^*WT*^‐TG mice; n=5). **G**, Na^+^‐K^+^‐ATPase activity in primary cultures of RPT cells from *hD*_*5*_ *R* ^*WT*^‐TG and *hD*_*5*_ *R* ^*F173L*^‐TG mice (*P<0.05 compared with *hD*_*5*_ *R* ^*WT*^‐TG mice; n=11). GAPDH indicates glyceraldehyde‐3‐phosphate dehydrogenase; *hD*_*5*_ *R* ^*F173L*^ –TG, human dopamine D_5_ receptor mutant F173L transgenic; *hD*_*5*_ *R* ^*WT*^‐TG, human dopamine D_5_ receptor wild‐type transgenic; RPT, renal proximal tubule; Trx1, thioredoxin 1.

Furthermore, we measured expression of genes encoding proteins involved in regulation or production of ROS, including *Sod‐1*,* Cat*,* Gpx‐1*,* Trx1*, and *Pparγ*, in kidneys of *hD*_*5*_ *R* ^*F173L*^‐TG mice and *hD*_*5*_ *R* ^*WT*^‐TG mice using real‐time quantitative PCR. Among these genes, *Trx1* attracted our attention because it has a central function in maintenance of redox homeostasis in cells and because *Trx1* mRNA level decreased to the greatest extent in *hD*_*5*_ *R* ^*F173L*^‐TG mice, relative to other genes studied (Figure 4A and Figure S3A through 3D). Respective levels of mRNA changes of 5 genes were as follows: 46% reduction in *Trx1*, 21% reduction in *Sod‐1*, 35% reduction in *Cat*, 31% reduction in *Gpx‐1*, and 87% increase in *Ppar*γ. Decreased *Trx1* mRNA expression was also associated with decreased Trx1 protein abundance in *hD*_*5*_ *R* ^*F173L*^‐TG mice, as quantified by immunoblotting (Figure 4B).

To study regulation of Trx1 expression by D_5_R, *hD*_*5*_ *R* ^*F173L*^ and *hD*_*5*_ *R* ^*WT*^ were expressed separately in mouse RPT cells. Successful transfection was confirmed by immunoblotting (Figure S4); that is, D_5_R protein expression was higher in transfected *hD*_*5*_ *R* ^*F173L*^ and *hD*_*5*_ *R* ^*WT*^ cells than in control cells transformed with the empty vector, but did not differ between *hD*_*5*_ *R* ^*F173L*^ and *hD*_*5*_ *R* ^*WT*^ cells. Consistent with in vivo results, both *Trx1* mRNA and protein expressions were lower in *hD*_*5*_ *R* ^*F173L*^‐transfected cells than in *hD*_*5*_ *R* ^*WT*^‐transfected cells (Figure 4C and 4D). We next investigated the effect of exogenous recombinant human Trx1 protein on Na^+^‐K^+^‐ATPase activity in *hD*_*5*_ *R* ^*F173L*^‐transfected and *hD*_*5*_ *R* ^*WT*^‐transfected cells. Compared with *hD*_*5*_ *R* ^*WT*^‐transfected cells, Na^+^‐K^+^‐ATPase activity was greater in *hD*_*5*_ *R* ^*F173L*^‐transfected cells and was returned to normal by treatment with exogenous recombinant human Trx1 protein (Figure 4E).

Results obtained using transfected mouse RPT cells were corroborated by studies using primary cultures of RPT cells from *hD*_*5*_ *R* ^*F173L*^‐TG and *hD*_*5*_ *R* ^*WT*^‐TG mice (Figure S5). Results showed that Trx1 expression was lower, but Na^+^‐K^+^‐ATPase activity was higher in RPT cells from *hD*_*5*_ *R* ^*F173L*^‐TG mice than in those from *hD*_*5*_ *R* ^*WT*^‐TG mice (Figure 4F and 4G).

### Role of Hyperphosphorylated D_5_R in Dysregulation of Trx1 in *hD*_*5*_ *R* ^*F173L*^‐TG Mouse Kidney

**Figure 5:**
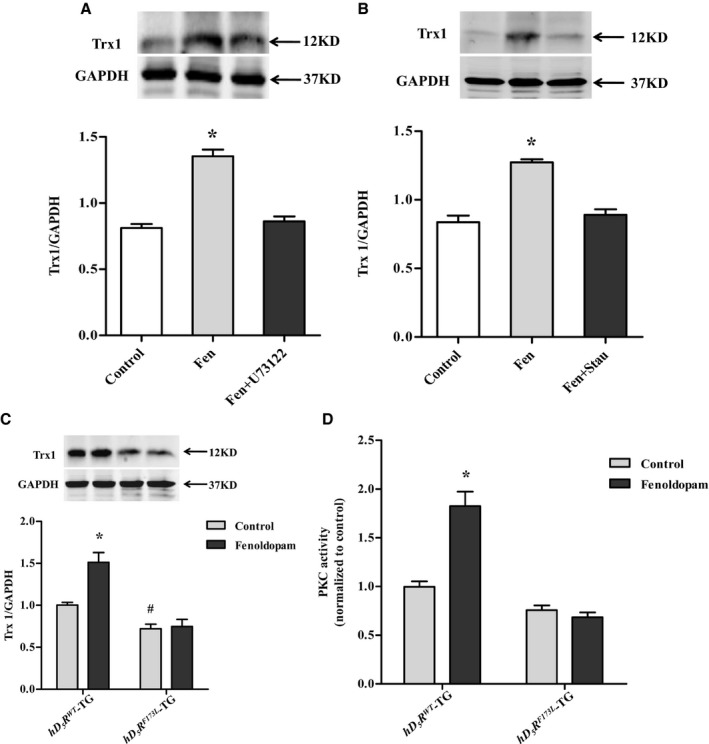
Mechanism of renal D_5_R regulation of Trx1 expression. **A**, Role of PLC in regulation of Trx1 expression by D_5_R. U73122, a PLC inhibitor, blocked the stimulatory effect of fenoldopam on Trx1 expression in primary cultures of RPT cells from *D1dr* knockout mice (*P<0.05 compared with other treatments; n=6; Fen: 10^−6 ^mol/L, 24 hours; U73122: 10^−6^ mol/L). **B**, Role of PKC in regulation of Trx1 expression by D_5_R. Staurosporine, a PKC inhibitor, blocked the stimulatory effect of Fen on Trx1 expression in primary cultures of RPT cells from *D1dr* knockout mice. (*P<0.05 compared with others; n=6; Fen: 10^−6^ mol/L, 24 hours; Stau: 10^−8^ mol/L). **C**, Trx1 expression in primary cultures of RPT cells from *hD*_*5*_ *R* ^*WT*^‐TG and *hD*_*5*_ *R* ^*F173L*^‐TG mice treated with Fen (*P<0.05 compared with control; ^#^ *P*<0.05 compared with *hD*_*5*_ *R* ^*WT*^‐TG; n=6; Fen: 10^−6^ mol/L, 24 hours). **D**, PKC activity in primary cultures of RPT cells from *hD*_*5*_ *R* ^*WT*^‐TG and *hD*_*5*_ *R* ^*F173L*^‐TG mice treated with Fen (*P<0.05 compared with other treatments; n=3; Fen: 10^−6^ mol/L, 30 minutes). D_5_R indicates dopamine D_5_ receptor; *D1dr,* dopamine D_1_ receptor; Fen, fenoldopam; GAPDH, glyceraldehyde‐3‐phosphate dehydrogenase; *hD*_*5*_ *R* ^*F173L*^, human dopamine D_5_ receptor mutant F173L; *hD*_*5*_ *R* ^*WT*^, human dopamine D_5_ receptor wild type; PKC, protein kinase C; PLC, phospholipase C; RPT, renal proximal tubule; Stau, staurosporine; Trx1, thioredoxin 1.

Studies have shown that D_5_R signal transduction is mediated, in part, by the phospholipase C (PLC)/PKC pathway.^32^, ^33^, ^34^ However, it is not known whether renal D_5_R‐mediated regulation of Trx1 occurs through the PLC/PKC signal pathway. Because no commercially available agonists can distinguish activity of D_5_R from that of D_1_R, we used RPT cells cultured from D_1_R knockout mice to study the effect of fenoldopam on D_5_R function (Figure S6). We found that the D_1_R/D_5_R agonist, fenoldopam (10^−6 ^mol/L, 24 hours), which is a D_5_R agonist in the absence of D_1_R, increased Trx1 expression that was inhibited by U73122 (10^−6^ mol/L; PLC inhibitor) and staurosporine (10^−8 ^mol/L; PKC inhibitor) in RPT cells from D_1_R knockout mice (Figure 5A and 5B). These data indicate that the PLC/PKC pathway is involved in positive regulation of Trx1 expression by D_5_R in RPT cells.

Consistent with results presented from primary cultures of RPT cells from D_1_R knockout mice, fenoldopam increased Trx1 expression in RPT cells from *hD*_*5*_ *R* ^*WT*^‐TG mice, but not in RPT cells from *hD*_*5*_ *R* ^*F173L*^‐TG mice (Figure 5C). Fenoldopam increased PKC activity in RPT cells from *hD*_*5*_ *R* ^*WT*^‐TG mice, but not in RPT cells from *hD*_*5*_ *R* ^*F173L*^‐TG mice (Figure 5D).

**Figure 6:**
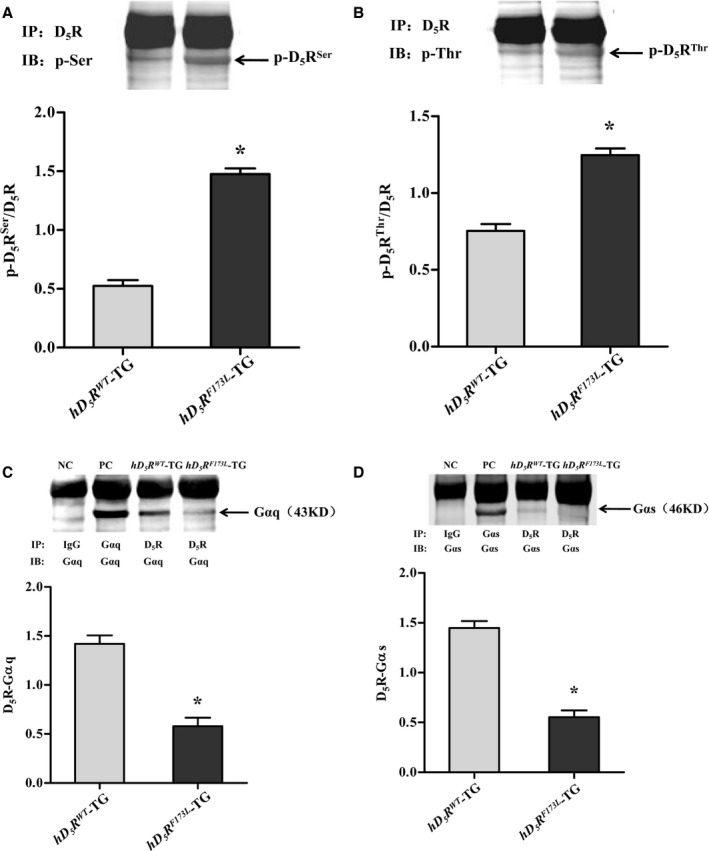
Phosphorylation of renal D_5_R and its association with Gαs/Gαq in *hD*_*5*_ *R* ^*WT*^‐TG and *hD*_*5*_ *R* ^*F173L*^‐TG mice. A and B, Serine (**A**) and threonine (**B**) phosphorylation of D_5_R in *hD*_*5*_ *R* ^*WT*^‐TG and *hD*_*5*_ *R* ^*F173L*^‐TG mice. Samples were immunoprecipitated with anti‐D_5_R antibody and immunoblotted with anti‐phosphoserine or ‐phosphothreonine antibody (*P<0.05 compared with *hD*_*5*_ *R* ^*WT*^‐TG mice; n=6). **C** and **D**, Coimmunoprecipitation of D_5_R and Gαq (C) or Gαs (**D**) in kidney homogenates from *hD*_*5*_ *R* ^*WT*^‐TG and *hD*_*5*_ *R* ^*F173L*^‐TG mice. Homogenates were immunoprecipitated with anti‐D_5_R antibody and immunoblotted with anti‐Gαq or anti‐Gαs antibody. For positive control, anti‐Gαq or ‐Gαs antibody was used instead of anti‐D_5_R antibody for immunoprecipitation, and for negative control (NC), IgG was used instead of anti‐D_5_R antibody for immunoprecipitation (*P<0.05 compared with *hD*_*5*_ *R* ^*WT*^‐TG mice; n=5 or 6). D_5_R indicates dopamine D_5_ receptor; Gαs, G protein alpha s subunit; Gαq, G protein alpha q subunit; *hD*_*5*_ *R* ^*F173L*^ ‐TG, human dopamine D_5_ receptor mutant F173L transgenic; *hD*_*5*_ *R* ^*WT*^‐TG, human dopamine D_5_ receptor wild‐type transgenic; IB, immunoblotting; IgG, immunoglobulin G; IP, immunoprecipitation; NC, negative control; PC, positive control; Ser, serine; Thr, threonine.

Hyperphosphorylation of D_1_R in hypertension leads to its dysfunction.^35^, ^36^ Similarly, our present study also showed that D_5_R was hyperphosphorylated in *hD*_*5*_ *R* ^*F173L*^‐TG mice (Figure 6A and 6B). As compared with *hD*_*5*_ *R* ^*WT*^‐TG mice, coimmunoprecipitation of hyperphosphorylated D_5_R with either Gαs or Gαq was decreased in kidneys of *hD*_*5*_ *R* ^*F173L*^‐TG mice (Figure 6C and 6D). Gαs and Gαq operate upstream of PLC‐PKC.^37^, ^38^ Therefore, we suggest that the hD_5_R^F173L^ mutation causes hyperphosphorylation of D_5_R, which leads to its dissociation from both Gαs and Gαq, subsequently impairs the D_5_R/PLC/PKC pathway, and prevents positive regulation of Trx1 expression by D_5_R in kidney.

## Discussion

D_1_‐like dopamine receptors play an important role in regulation of blood pressure.^5^, ^6^, ^7^, ^8^, ^9^, ^14^, ^15^, ^16^, ^17^, ^18^, ^19^ D_5_R has higher affinity for dopamine than D_1_R and exhibits constitutive activity.^9^, ^10^, ^11^ Basal cAMP accumulation is greater in HEK293 cells expressing human D_5_R than in HEK293 cells expressing human D_1_R.^32^, ^39^ However, D_1_R increases cAMP production to a greater extent than D_5_R when these proteins are expressed separately, but in the same quantities in HEK293 cells.^32^, ^39^ In RPT cells, D_1_R is associated with both adenylyl cyclase and PLC activities, whereas D_5_R is associated mainly with PLC. This contrasts with the association of D_5_R and adenylyl cyclase activities when D_5_R is heterologously expressed in HEK293 cells.^32^, ^39^ More recently, studies have focused on the role of D_5_R in regulation of blood pressure. Disruption of the *Drd5* gene results in hypertension in *Drd5* ^*−/−*^ mice, and a high‐salt diet further aggravates elevated blood pressure in these mice.^14^, ^15^, ^16^, ^17^, ^18^, ^19^ In the RPT, D_5_R, but not D_1_R, positively regulates expression of renalase, which is involved in degradation of epinephrine, which normally increases renal sodium transport.^40^ D_5_R, but not D_1_R, is also responsible for degradation of AT_1_R in RPT cells.^14^, ^41^ D_5_R and the gastrin receptor synergistically interact in kidney to maintain normal sodium balance following an increase in sodium intake.^42^ Thus, renal D_5_R, by itself, or through interactions with other G‐protein‐coupled receptors, plays an important role in regulation of renal sodium transport and blood pressure.

The human gene encoding D_5_R, the *DRD5* locus at 4p15.1 to 16.1, is associated with essential hypertension^12^, ^13^ and metabolic syndrome, of which hypertension is a component.^43^ Single‐nucleotide polymorphisms in, and duplications of, the *DRD5* gene have been found in humans,^21^, ^22^, ^23^ and carriers of the hD_5_R^F173L^ mutation have a decreased ability to stimulate cAMP production.^17^, ^24^ Therefore, to investigate the role of D_5_R in the elaboration of hypertension, we generated *hD*_*5*_ *R* ^*F173L*^‐TG and *hD*_*5*_ *R* ^*WT*^‐TG mice. In the present study, we found that *hD*_*5*_ *R* ^*F173L*^‐TG mice developed hypertension with decreased urine flow and sodium excretion, which might be partly attributed to an increase in Na^+^‐K^+^‐ATPase activity in the RPT. Moreover, we showed that fenoldopam, an agonist of D_1_‐like receptors, increased urine flow and sodium excretion to a greater extent in *hD*_*5*_ *R* ^*WT*^‐TG mice than in *hD*_*5*_ *R* ^*F173L*^‐TG mice, suggesting dysfunction of D_5_R in *hD*_*5*_ *R* ^*F173L*^‐TG mice. We also found that infusion of the varying doses of fenoldopam did not affect blood pressures in either *hD*_*5*_ *R* ^*F173L*^‐TG or *hD*_*5*_ *R* ^*WT*^‐TG mice, which means that the differential effects of fenoldopam on urine flow and sodium excretion in these 2 groups of mice were not caused by differential effects on blood pressure.

It should be noted that D_5_Rs are not only expressed in the proximal convoluted and straight tubules (including brush border membranes), but also in thick ascending limb of Henle, distal convoluted tubule, and cortical and outer medullary collecting ducts.^16^ Moreover, disruption of D_5_R gene causes increased expression of renal sodium transporter, channels, and pump in mice, for example: D_5_R‐deficient mice have greater renal protein expressions of NKCC2 (sodium‐potassium‐2 chloride cotransporter), NCC (sodium chloride cotransporter), and α and γ subunits of ENaC (epithelial sodium channel) on control and elevated sodium diet; however, expression of the proximal sodium transporters, NHE3 (sodium hydrogen exchanger type 3) and NaPi2 (sodium phosphate cotransporter type 2), is increased only on elevated sodium diet.^16^ Therefore, the attenuated natriuretic capacity of *hD*_*5*_ *R* ^*F173L*^‐TG mice is also possibly caused by dysregulation of D_5_R on other nephron segments and sodium transporter, channels, and pump.

The effect of D_5_R on blood pressure can be modulated through its ability to regulate oxidative stress. Activation of D_5_R has been reported to decrease oxidative stress in kidney through a phospholipase D–mediated signal transduction pathway.^19^ Compared with wild‐type mice, *Drd5* ^−/−^ mice have increased expression and activity of gp91phox, p47phox, and Nox 4 in the kidney.^17^ Our present study also showed that both whole body and renal oxidative stress were increased in *hD*_*5*_ *R* ^*F173L*^‐TG mice.

Trx1 is a 12‐kDa protein that regulates signaling molecules in redox‐regulated gene pathways.^44^, ^45^, ^46^, ^47^ Trx1 is a cytosolic thiol antioxidant and redox‐active protein that plays a vital role in maintenance of the intracellular redox state.^48^ There is some evidence that Trx1 is involved in the pathogenesis of cardiovascular diseases, including hypertension. For example, *Trx1* mRNA levels are increased in mononuclear cells from hypertensive humans, but are decreased after 3 months of antihypertensive treatment.^49^ Angiotensin II–induced high blood pressure has been associated with a 3‐fold increase in cardiac Trx expression in mice.^50^ However, Trx expression is decreased in aorta, heart, and kidney of the spontaneously hypertensive rat relative to the normotensive Wistar–Kyoto rat. Moreover, angiotensin II is less able to increase Trx expression in peripheral blood mononuclear cells of the spontaneously hypertensive rat.^51^ When adenovirus carrying the coding sequence of the *Trx1* gene was injected into the left ventricle of the spontaneously hypertensive rat 48 hours before ligation of the left anterior descending coronary artery, infarct size, number of apoptotic cardiomyocytes, and left ventricular inner diameter decreased and the ejection fraction and fractional shortening increased.^52^ In the present study, we showed that *Trx1* gene and protein expression were decreased in both renal homogenates and RPT cells of *hD*_*5*_ *R* ^*F173L*^‐TG mice. Furthermore, we found that renal Na^+^‐K^+^‐ATPase activity was higher in *hD*_*5*_ *R* ^*F173L*^ RPT cells than in *hD*_*5*_ *R* ^*WT*^ RPT cells, but that the former could be normalized by treatment with exogenous recombinant human Trx1 protein.

Activity of dopamine receptors is regulated by phosphorylation/dephosphorylation.^5^, ^6^, ^7^, ^25^, ^26^, ^53^ In hypertension, uncoupling of D_1_R from its G protein/effector complex in RPT is caused, in part, by increased D_1_R phosphorylation^25^, ^26^, ^53^ that impairs D_1_R function, increases production of second messengers, and inhibits the activity of sodium transporters/pump.^4^, ^5^, ^6^, ^7^ Until now, the roles of phosphorylation and uncoupling of D_5_R from its G protein/effector complex in the development of hypertension have not been studied. Similar to D_1_R, D_5_R is coupled to the stimulatory Gα‐subunit, Gαs, and stimulates adenylyl cyclase activity. However, D_5_R may be also coupled to Gαq.^32^, ^33^, ^34^, ^54^ Our present studies showed that, compared with *hD*_*5*_^*WT*^‐TG mice, phosphorylation of renal D_5_R was increased in *hD*_*5*_ *R* ^*F173L*^‐TG mice, which consequently led to dissociation of D_5_R from both Gαs and Gαq. Furthermore, we used primary RPT cells from D_1_R knockout mice and *hD*_*5*_ *R* ^*F173L*^‐TG mice to determine the effect of fenoldopam on D_5_R function because there is no commercially available agonist that can distinguish the activities D_1_R or D_5_R. Our results in RPT cells from D_1_R knockout mice using the PLC inhibitor, U73122, and the PKC inhibitor, staurosporine, also showed that the PLC/PKC pathway is involved in D_5_R‐mediated regulation of renal Trx1 expression. This suggests that aberrant D_5_R regulation of PLC/PKC signaling in *hD*_*5*_ *R* ^*F173L*^‐TG mice might play an important role in the pathogenesis of hypertension.

Studies have shown that there are roles for the D_5_R in other organs beside the kidney. For example: mice lacking D_5_R are hypertensive, which is attributable to increased sympathetic tone, and consequently decreases natriuresis and diuresis; D_5_R knockout mice also have cardiac hypertrophy and increased heart weight; D_5_R has also shown to exert antiproliferative, ‐migration, and ‐oxidative effects on vascular smooth muscle cells.^55^, ^56^, ^57^, ^58^ Thus, we can presume that D_5_Rs in the central nervous system, heart, and vasculature may interact with D_5_Rs in the kidney. A recent study showed that overexpression of a cardiac‐specific hD_5_R^F173L^ in mice causes a dilated cardiomyopathy through ROS overgeneration by nicotinamide adenine dinucleotide phosphate oxidase activation and nuclear factor‐like 2 degradation, which showed similar results with that in D_5_R‐deficient mice.^59^ Therefore, we speculate that there may also be interactions between these hD_5_R^F173L^ in the kidney and other sites, such as the central nervous system, heart, and vasculature, which needs to be confirmed in the future.

In summary, we showed that *hD*_*5*_ *R* ^*F173L*^‐TG mice have hypertension with impaired urine flow and sodium excretion. Impaired renal function of *hD*_*5*_ *R* ^*F173L*^‐TG mice might be related to increased oxidative stress in the kidney. Our study suggests that increased blood pressure in *hD*_*5*_ *R* ^*F173L*^‐TG mice is related to decreased Trx1 expression in the kidney. Hyperphosphorylation of D_5_R in *hD*_*5*_ *R* ^*F173L*^‐TG mice, which leads to dissociation of D_5_R from Gαs and Gαq, appears to be a key factor that impairs D_5_R function.

## Sources of Funding

This study was supported, in part, by grants from the National Key R&D Program of China (2018YFC1312700), the National Natural Science Foundation of China (81100500, 81570379), the Program of Innovative Research Team by National Natural Science Foundation (81721001), Program for Changjiang Scholars and Innovative Research Team in University (IRT1216), and the National Institutes of Health (5R01DK039308‐31, 7R37HL023081‐37, and 5P01HL074940‐11).

## Disclosures

None.

## Supporting information


**Table S1.** PCR Primers for Amplifying Mouse *Sod‐1*,* Cat*,* Gpx‐1*,* Pparγ*, and *Trx1* Sequences
**Table S2.** Sodium, Water, and Food Intakes in Human Dopamine D5 Receptor Wild‐Type Transgenic (*hD5RWT*‐TG) and Human Dopamine D5 Receptor Mutant F173L Transgenic (*hD5RF173L*‐TG) Mice
**Figure S1**. Genotypic difference and their identification of transgenic mice. Genotypic difference between *hD5RF173L*‐TG and *hD5RWT*‐TG mice. A, The genotypes of mice used in the present study were confirmed by PCR (B). Lane W: vector control (C57BL/6 mice); Lane N: negative control (negative control C57BL/6 mice generated by microinjection of empty plasmid constructs); Lane P: positive control (positive control C57BL/6 mice were generated by microinjection of the pcDNA2*hD5RWT* construct); Lane M: DNA molecular size marker; Lanes 1 to 3: DNA samples from *hD5RWT*‐TG mice; lanes 4 to 6: DNA samples from *hD5RF173L*‐TG mice. *hD5RF173L*‐TG indicates human dopamine D5 receptor mutant F173L transgenic; *hD5RWT*‐TG, human dopamine D5 receptor wild‐type transgenic.
**Figure S2**. Expression of D5R in *hD5RWT*‐TG and *hD5RF173L*‐TG in mouse kidney. Expression of D5R protein in C57BL/6 mice, *hD5RWT*‐TG, and *hD5RF173L*‐TG were detected in mice by immunoblotting. Results are expressed as the ratio of the density of D5R to GAPDH (n=6; ^*^ *P*<0.05 compared with C57BL/6 mice). D5R indicates dopamine D5 receptor; GAPDH, glyceraldehyde‐3‐phosphate dehydrogenase; *hD5RF173L*‐TG, human dopamine D5 receptor mutant F173L transgenic; *hD5RWT*‐TG, human dopamine D5 receptor wild‐type transgenic.
**Figure S3.** Expression of reactive oxygen species–related genes in *hD5RWT*‐TG and *hD5RF173L*‐TG mice. Expression of *Sod‐1* (A), *Cat* (B), *Gpx‐1* (C), and *Pparγ* (D) mRNAs in *hD5RWT*‐TG and *hD5RF173L*‐TG mice was measured using qRT‐PCR (^*^ *P*<0.05 compared with *hD5RWT*‐TG mice; n=8). *Cat* indicates catalase; *Gpx‐1*, glutathione peroxidase 1; *hD5RF173L*‐TG, human dopamine D5 receptor mutant F173L transgenic; *hD5RWT*‐TG, human dopamine D5 receptor wild‐type transgenic; *Pparγ*, peroxisome proliferator‐activated receptor gamma; qRT‐PCR, real‐time quantitative polymerase chain reaction; *Sod‐1*, superoxide dismutase 1.
**Figure S4.** Identification of *hD5RWT* and *hD5RF173L* transfected into mouse RPT cells. Successful *hD5RWT* and *hD5RF173L* transfections into mouse RPT cells were verified by immunoblotting (^*^ *P*<0.05 compared with *hD5RWT*; n=6). D5R indicates dopamine D5 receptor; *hD5RF173L*, human dopamine D5 receptor mutant F173L; *hD5RWT*, human dopamine D5 receptor wild type; RPT, renal proximal tubule.
**Figure S5**. Identification of mouse RPT cells in primary culture. Red fluorescence: megalin (RPT cell marker); blue fluorescence: nucleus (DAPI). DAPI indicates 4′,6‐diamidino‐2‐phenylindole; RPT, renal proximal tubule.
**Figure S6.** Expression of dopamine D1 receptor in *D1dr* knockout mice. Expression of D1R protein in *D1dr* knockout and *D1dr* wild‐type mice was quantified by immunoblotting. D1R indicates dopamine D1 receptor; *D1dr*, dopamine D1 receptor; KO, knockout; WT, wild type.
