# Supplementary material for: Role of Thioredoxin 1 in Impaired Renal Sodium Excretion of hD 5 R F173L Transgenic Mice
Source: J Am Heart Assoc. 2019 Apr 6;8(8):e012192. doi: 10.1161/JAHA.119.012192 (PMC6507211; doi:10.1161/JAHA.119.012192)
Supplement: Supplementary file 1 — Table S1. PCR Primers for Amplifying Mouse Sod‐1, Cat, Gpx‐1, Pparγ, and Trx1 Sequences Table S2. Sodium, Water, and Food Intakes in Human Dopamine D5 Receptor Wild‐Type Transgenic (hD5RWT‐TG) and Human Dopamine D5 Receptor Mutant F173L Transgenic (hD5RF173L‐TG) Mice Figure S1. Genotypic difference and their identification of transgenic mice. Genotypic difference between hD5RF173L‐TG and hD5RWT‐TG mice. A, The genotypes of mice used in the present study were confirmed by PCR (B). Lane W: vector control (C57BL/6 mice); Lane N: negative control (negative control C57BL/6 mice generated by microinjection of empty plasmid constructs); Lane P: positive control (positive control C57BL/6 mice were generated by microinjection of the pcDNA2hD5RWT construct); Lane M: DNA molecular size marker; Lanes 1 to 3: DNA samples from hD5RWT‐TG mice; lanes 4 to 6: DNA samples from hD5RF173L‐TG mice. hD5RF173L‐TG indicates human dopamine D5 receptor mutant F173L transgenic; hD5RWT‐TG, human dopamine D5 receptor wild‐type transgenic. Figure S2. Expression of D5R in hD5RWT‐TG and hD5RF173L‐TG in mouse kidney. Expression of D5R protein in C57BL/6 mice, hD5RWT‐TG, and hD5RF173L‐TG were detected in mice by immunoblotting. Results are expressed as the ratio of the density of D5R to GAPDH (n=6; * P<0.05 compared with C57BL/6 mice). D5R indicates dopamine D5 receptor; GAPDH, glyceraldehyde‐3‐phosphate dehydrogenase; hD5RF173L‐TG, human dopamine D5 receptor mutant F173L transgenic; hD5RWT‐TG, human dopamine D5 receptor wild‐type transgenic. Figure S3. Expression of reactive oxygen species–related genes in hD5RWT‐TG and hD5RF173L‐TG mice. Expression of Sod‐1 (A), Cat (B), Gpx‐1 (C), and Pparγ (D) mRNAs in hD5RWT‐TG and hD5RF173L‐TG mice was measured using qRT‐PCR (* P<0.05 compared with hD5RWT‐TG mice; n=8). Cat indicates catalase; Gpx‐1, glutathione peroxidase 1; hD5RF173L‐TG, human dopamine D5 receptor mutant F173L transgenic; hD5RWT‐TG, human dopamine D5 receptor wild‐type transgenic; Pparγ, per [file JAH3-8-e012192-s001.pdf]

SUPPLEMENTAL MATERIAL

Table S1. PCR primers for amplifying mouse *Sod-1*, *Cat*, *Gpx-1*, *Pparγ*, and *Trx1* sequences.

Gene Name	Primers	Temperature conditions
Sod-1	Forward: 5'-CTCGTCTTGCTCTCTCTGGTC-3<br>Reverse: 5'-CTTCTGCTCGAAGTGGATGG-3'	95 °C 10 s<br>60 °C 20 s<br>68 °C 1 min
Cat	Forward: 5'-GCAGATACCTGTGAACTGTC-3'<br>Reverse: 5'-GTAGAATGTCCGCACCTGAG-3'	95 °C 10 s<br>54 °C 20 s<br>68 °C 1 min
Gpx-1	Forward: 5'-CCTCAAGTACGTCCGACCTG-3'<br>Reverse: 5'-CAATGTCGTTGCGGCACACC-3'	95 °C 10 s<br>54 °C 20 s<br>68 °C 1 min
Pparγ	Forward: 5'-TTTCAAGGGTGCCAGTTT-3'<br>Reverse: 5'-GGAGGTCAGCATCGTGTAG-3'	95 °C 10 s<br>54 °C 20 s<br>68 °C 1 min
Trx1	Forward:5'-GGTGTGGACCTTGCAAAATGATC-3'<br>Reverse: 5'--GGCTTCAAGCTTTTCCTT-3'	95 °C 10 s<br>60 °C 20 s<br>68 °C 1 min
Gapdh	Forward: 5'-GGCCTCCAAGGAGTAAGAAA-3'<br>Reverse: 5'-GCCCCTCCTGTTATTATGG-3'	95 °C 10 s<br>60 °C 20 s<br>68 °C 1 min

Cat indicates catalase; *Gapdh*, glyceraldehyde-3-phosphate dehydrogenase; *Gpx-1*, glutathione peroxidase 1; PCR, polymerase chain reaction; *Pparγ*, peroxisome proliferator activated receptor gamma; *Sod-1*, superoxide dismutase 1; *Trx1*, thioredoxin 1.

Table S2. Sodium, water and food intakes in human dopamine D_5 receptor wild type transgenic (*hD_5R^{WT}*-TG) and human dopamine D_5 receptor mutant F173L transgenic (*hD_5R^{F173L}*-TG) mice.

Age (4 months)	NaCL intake (mg/day)	Water intake (ml/day)	Food intake （g/day）
hD_5R^{WT}-TG	17.83±3.52	3.42±0.75	3.47±0.74
hD_5R^{F173L}-TG	17.67±3.41	3.33±0.78	3.43±0.71

Figure S1. **Genotypic difference and their identification of transgenic mice. Genotypic difference between *hD₅R*^{F173L}-TG and *hD₅R*^{WT}-TG mice.**

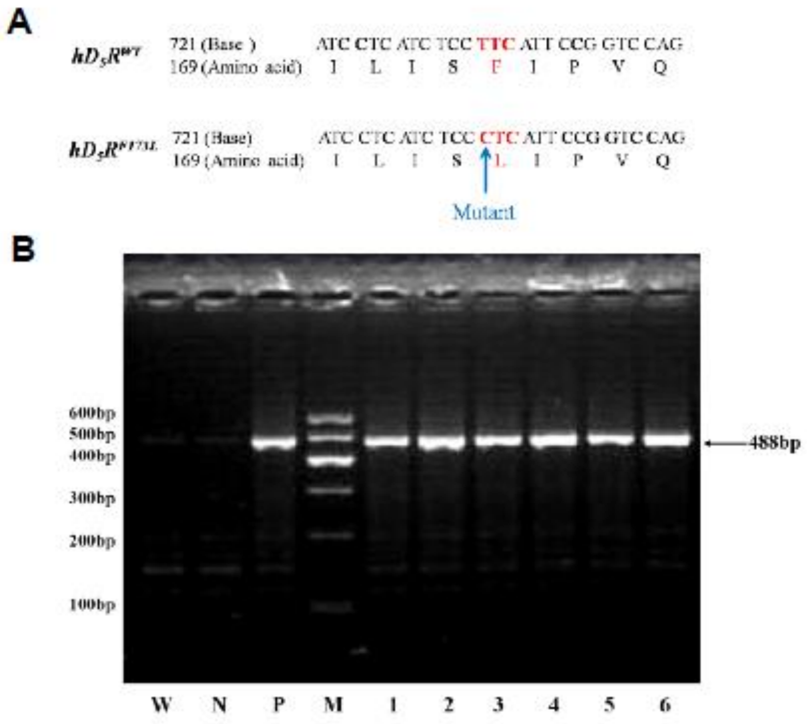

(A). The genotypes of mice used in the present study were confirmed by PCR (B). Lane W: vector control (C57BL/6 mice); Lane N: negative control (negative control C57BL/6 mice generated by microinjection of empty plasmid constructs); Lane P: positive control (positive control C57BL/6 mice were generated by microinjection of the pcDNA2*hD*$_5$*R*^{WT} construct); Lane M: DNA molecular size marker; Lanes 1-3: DNA samples from *hD*$_5$*R*^{WT}-TG mice; lanes 4-6: DNA samples from *hD*$_5$*R*^{F173L}-TG mice. *hD*$_5$*R*^{F173L} -TG indicates human dopamine D$_5$ receptor mutant F173L transgenic; *hD*$_5$*R*^{WT}-TG, human dopamine D$_5$ receptor wild type transgenic.

Figure S2. **Expression of D_5R in *hD_5R^{WT}*-TG and *hD_5R^{F173L}*-TG in mouse kidney. Expression of D_5R protein in C57BL/6 mice, *hD_5R^{WT}*-TG, and *hD_5R^{F173L}*-TG were detected in mice by immunoblotting.**

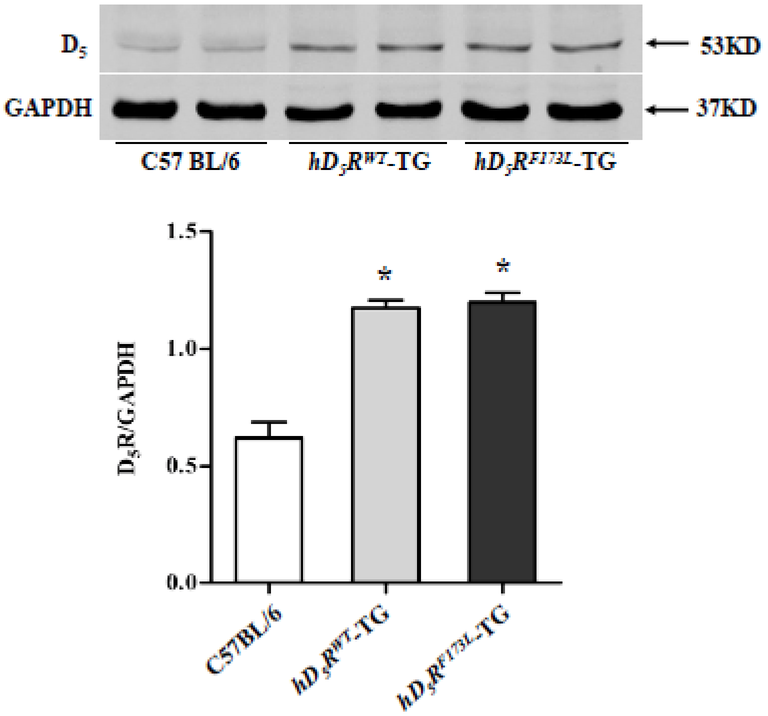

Results are expressed as the ratio of the density of D_5R to GAPDH (n = 6, *$P < 0.05$ compared with C57BL/6 mice). D_5R indicates dopamine D_5 receptor; GAPDH, glyceraldehyde-3-phosphate dehydrogenase; *hD_5R^{F173L}*-TG, human dopamine D_5 receptor mutant F173L transgenic; *hD_5R^{WT}*-TG, human dopamine D_5 receptor wild type transgenic.

Figure S3. Expression of reactive oxygen species-related genes in hD_5R^{WT}-TG and hD_5R^{F173L}-TG mice.

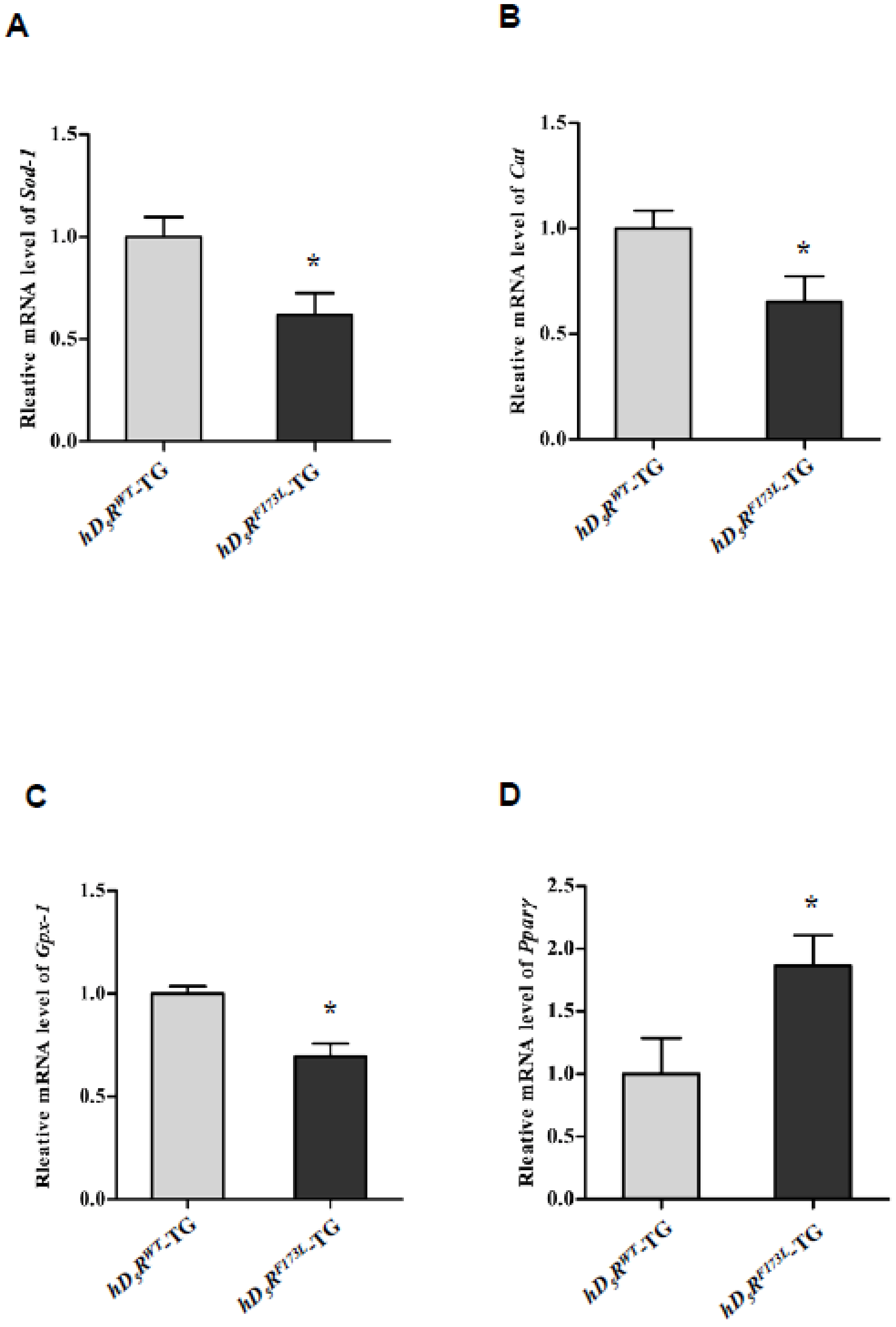

Expression of *Sod-1* (A), *Cat* (B), *Gpx-1* (C), and *Pparγ* (D) mRNAs in hD_5R^{WT}-TG and hD_5R^{F173L}-TG mice was measured using qRT-PCR (**P* < 0.05 compared with hD_5R^{WT}-TG mice, n = 8). *Cat* indicates catalase; *Gpx-1*, glutathione peroxidase 1; hD_5R^{F173L}-TG, human dopamine D_5 receptor mutant F173L transgenic; hD_5R^{WT}-TG, human dopamine D_5 receptor wild type transgenic; *Pparγ*, peroxisome proliferator activated receptor gamma; qRT-PCR, real-time quantitative polymerase chain reaction; *Sod-1*, superoxide dismutase 1.

Figure S4. Identification of *hD_5R^{WT}* and *hD_5R^{F173L}* transfected into mouse RPT cells.

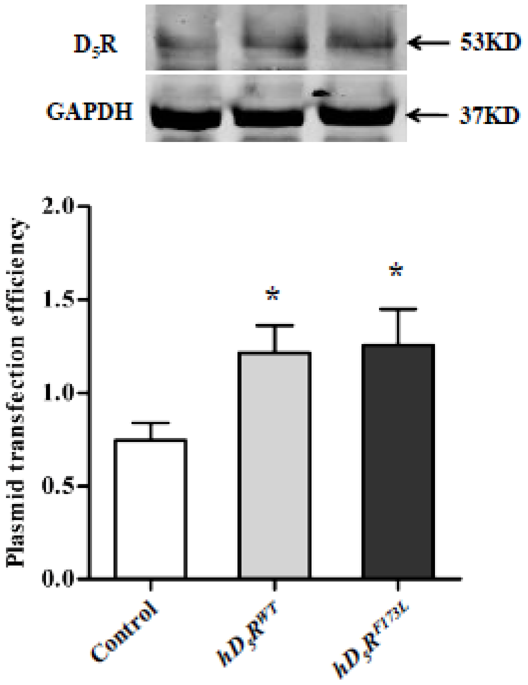

Successful hD_5R^{WT} and hD_5R^{F173L} transfections into mouse RPT cells were verified by immunoblotting (*$P < 0.05$ compared with hD_5R^{WT}, n = 6). D_5R indicates dopamine D_5 receptor; hD_5R^{F173L}, human dopamine D_5 receptor mutant F173L; hD_5R^{WT}, human dopamine D_5 receptor wild type; RPT, renal proximal tubule.

Figure S5. **Identification of mouse RPT cells in primary culture.**

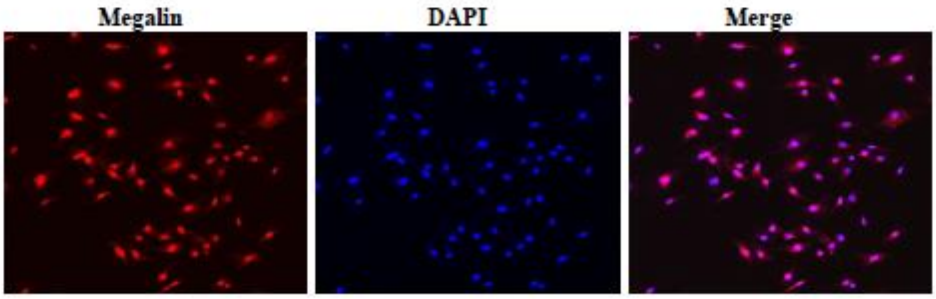

Red fluorescence: megalin (RPT cell marker); blue fluorescence: nucleus (DAPI). DAPI indicates 4’,6-diamidino-2-phenylindole; RPT, renal proximal tubule.

Figure S6. **Expression of dopamine D_1 receptor in *D1dr* knockout mice.**

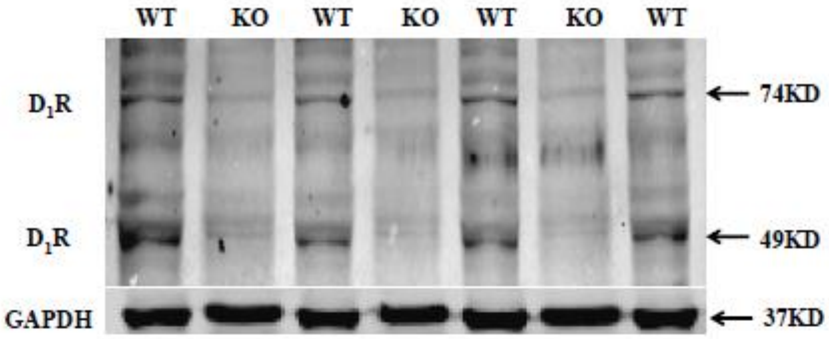

Expression of D_1R protein in *D1dr* knockout and *D1dr* wild-type mice was quantified by immunoblotting. D_1R indicates dopamine D_1 receptor; *D1dr,* dopamine D_1 receptor; KO, knockout; WT, wild-type.
